# A systematic review of complementary and alternative medicine in oncology: Psychological and physical effects of manipulative and body-based practices

**DOI:** 10.1371/journal.pone.0223564

**Published:** 2019-10-17

**Authors:** Nicolas Calcagni, Kamel Gana, Bruno Quintard

**Affiliations:** 1 Laboratoire EA 4136 / INSERM UMR 1219, Team: Handicap Activity Cognition Health (HACH), Bordeaux, France; 2 Univ. Bordeaux, Bordeaux, France; Beatrix Children’s Hospital, University Medical Center Groningen, NETHERLANDS

## Abstract

**Background:**

Complementary and Alternative Medicines (CAM) are widely used by cancer patients, despite limited evidence of efficacy. Manipulative and body-based practices are some of the most commonly used CAM. This systematic review evaluates their benefits in oncology.

**Method:**

A systematic literature review was carried out with no restriction of language, time, cancer location or type. PubMed, CENTRAL, PsycArticle, PsychInfo, Psychology and Behavioral Sciences Collection and SOCindex were queried. Inclusion criteria were adult cancer patients and randomized controlled trials (RCT) assessing manipulative and body-based complementary practices on psychological and symptom outcomes. Effect size was calculated when applicable.

**Results:**

Of 1624 articles retrieved, 41 articles were included: massage (24), reflexology (11), acupressure (6). Overall, 25 studies showed positive and significant effects on symptom outcomes (versus 9 that did not), especially pain and fatigue. Mixed outcomes were found for quality of life (8 papers finding a significant effect vs. 10 which did not) and mood (14 papers vs. 13). In most studies, there was a high risk of bias with a mean Jadad score of 2, making interpretation of results difficult.

**Conclusion:**

These results seem to indicate that manipulative CAM may be effective on symptom management in cancer. However, more robust methodologies are needed. The methodological requirements of randomized controlled trials are challenging, and more informative results may be provided by more pragmatic study design.

## Introduction

Given the physical and psychological distress induced by cancer treatments, there is consensus on the importance of considering psychosocial features for cancer patients [1–4]. Non-medicated therapies, known as “Complementary and Alternative Medicine” (CAM) [5], are becoming a more popular supportive care option. Several commonly used therapies are reflexology, osteopathy, or chiropractic care, coming under the heading of ‘manipulative and body-based practices’ [6]. However, the scientific evidence of their efficacy is still unclear.

The first problem may lie in lack of clarity and evolving definitions of Complementary and Alternative Medicine (CAM). An initial proposal by the World Health Organization defines: “a broad set of health care practices that are not part of that country’s own tradition and are not integrated into the dominant health care system” [5]. The National Center for Complementary and Integrative Health (NCCIH) and the Office of Cancer Complementary and Alternative Medicine (OCCAM) further categorize these practices that are not part of standard care into “complementary”: when the non-mainstream practice is used along with conventional medicine; “alternative”: when conventional medicine is replaced by the practice, and “integrative”: when conventional and complementary approaches are combined together in a coordinated way [7–10].

Another emerging term is given by the Collaborative university platform for Evaluating health Prevention and Supportive care Programs (CEPS), which suggests the term of Non-Pharmacological Intervention (NPI), defining NPIs as “non-invasive and non-pharmacological interventions on human health based on science” with an “observable impact on health, quality of life, behavioral and socioeconomic indicators” [8]. This definition differs from the earlier proposals in that it insists on the scientific approach needed to be labeled as NPI. Several dozen therapies can be gathered under these definitions, such as acupuncture, herbs, reiki, reflexology, homeopathy, meditation, diets, yet there is a lack of consensus on their classifications which vary in length and complexity across countries and authorities [7, 9–11].

CAM is widely used, especially in the case of cancer, and prevalence is around 37.5% in France, 35.9% in Europe, or 40% in USA [12–15]. Patients hope that CAM may help them in curing the illness, improving their well-being, lessening side-effects of their treatments, improving their immune system to resist cancer and its treatments, or even prolonging their survival [11, 16, 17]. A number of systematic reviews have been conducted to assess the efficacy of CAM [15, 18–22]. Results from these studies are often mixed; some trials show positive effects of the practices and others show negative results. The fact that most of the studies have low quality methodology with a high risk of bias adds to the uncertainty of their effects. Moreover, many of these systematic reviews incorporate several CAM without distinguishing between them, focus on only one kind of cancer limiting generalization of results, or are outdated. Thus, the efficacy of these CAM approaches has yet to be clearly demonstrated to provide evidence-based recommendations to patients and oncologists.

The primary aim of this review was to systematically identify and collate randomized controlled trials investigating the effects of manipulative and body-based practices in oncological settings, allowing a clearer assessment of their efficacy. The secondary objective was to identify which specific manipulative and body-based practices are used in oncology. We chose to focus on these techniques because as they are based on touch, they could stimulate a sense of wellness and pleasure on a body that is hurt by the illness and its invasive treatment [23].

## Methods and materials

### Eligibility criteria

#### Population

Studies including adult (≥ 18 years) patients with a diagnosis of cancer, with no limitations regarding time since diagnosis or cancer location were included.

#### Intervention

CAM interventions in oncology were included if they were associated with manipulative and body-based practices such as massage, reflexology, chiropractic, osteopathy, naprapthy and shiatsu/tui na/acupressure [24–30].

#### Comparison group

Randomized Controlled Trials (RCT) that featured any comparison control group (usual care, placebo, sham practice, visit by staff) were selected. Any trials involving self-administered and/or tool-assisted interventions only (use of acupressure bands without the intervention of a practitioner, for example) were excluded.

#### Outcomes

Studies featuring quality of life, psychosocial, symptoms and side-effect-related outcomes were included, based on the rationale that the main expectation of people using CAM was to improve well-being and to reduce/manage symptoms. Outcomes varied from quality of life to mood (anxiety, depression, stress) and symptom management (nausea, fatigue, pain).

#### Study design

The search was restricted to RCTs because they represent the gold standard by which health care professionals make decisions about treatment effectiveness. All RCT designs were accepted (parallel, cross-over, cluster), to ensure full coverage.

### Information sources

We queried the following electronic databases: PubMed, Cochrane Central Register of Controlled Trials (CENTRAL), PsycArticle, Psychinfo, Psychology and Behavioral Sciences Collection and SOCindex with no limitations for publication date nor language. The search was last updated on September 2018.

### Search algorithm

Keywords included successively in all databases were: massag* AND cancer AND trial; reflexolog* OR foot massag* OR feet massag* AND cancer AND trial; chiropract* AND cancer AND trial; osteopath* AND cancer AND trial; naprapath* AND cancer AND trial; acupressure AND cancer AND trial. We subsequently withdrew the command “AND trial” for Cochrane CENTRAL, as we narrowed the results to “Trials”, excluding Cochrane reviews, Other reviews, Methods Studies, Technology Assessments, Economic Evaluations and Cochrane Groups. Shiatsu and Tui Na practices were not directly included in our search strategy because acupressure—which shares the same principles with these techniques—is more represented in the scientific literature [31]. The first author performed all databases searches.

### Study selection

Articles were systematically screened on their title and abstract, which had to contain words linked to cancer or oncology, “trial”, and words linked to manipulative and body-based practices (such as reflexology, massage, acupressure, shiatsu…) to be included in the full review of the article. Studies were included in the final review when criteria of eligibility were confirmed after reading the whole article: RCT design, adequate population, relevant intervention and outcome. They were excluded if the participants were below the age of 18 (irrelevant population), the intervention was not linked to manipulative practice or was only self-administered or tool-assisted or mixed with other techniques (irrelevant intervention). PRISMA guidelines were followed throughout the present review [32].

### Data items and collection process

Information collected from the selected studies was chosen according to the procedure initiated by McVicar et al. [20]. For each paper, information about setting of the studies (year of publication, country of publication, kind of cancer, intervention used, dose and duration of the sessions, percentage of female participant, sample size), method used (design of the RCT, degree of blinding, randomization method, control strategy) and results (significant outcome, non-significant outcomes, and where possible mean of each group before and after intervention) had to be systematically noted. These data were manually identified in each study and noted on an excel file that was used for calculations. Dual independent selection, inclusion, data extraction and comparison were performed by NC and BQ for the first 20 studies of each databases. KG was the referee in case of doubts or disagreement. If consensus was reached on these 20 studies, the rest of selection, exclusions and data extractions were shared between NC and BQ in a single fashion.

### Summary measure

Simple descriptive statistics and qualitative content analysis were used. As our studies showed clinical heterogeneity in terms of outcomes, kind of cancer, interventions, design, control strategy, implementation and timing of evaluations, it was impractical to run quantitative analysis for a meta-review. As such, we strengthened our analysis by calculating, where possible, the effect size and the correlated 95% Confidence Interval (95% CI) [33]. Effect sizes (Standardized Mean Difference) were calculated based on means (M) and standard deviations (SD) of intervention and control groups only [34]. When a study measured the same variable more than two times, a pooled effect size was obtained using the mean of a “meta-analysis-like” method with random effects model using Exploratory Software for Confidence Intervals (ESCI) [35]. Effect size was calculated before intervention (to verify absence of group effect before intervention) and after intervention. NC and KG performed the qualitative and quantitative analyses of the study.

### Risk of bias

The Jadad scale, which is a commonly used three-item five-point quality scale, was used to rate independently the quality of the trials and to allocate a score of between zero (very poor) and five (rigorous) [36]. Two points were given if the research used appropriate randomization (at least randomization by block with random variation). Two other points were allocated if the authors reported the use of double blinding. A last point was allocated if authors integrated description of the withdrawals in their study. To maximize study inclusions, we did not use a minimum cut-off score as an inclusion criterion.

## Results

### Study selection

**Fig 1:**
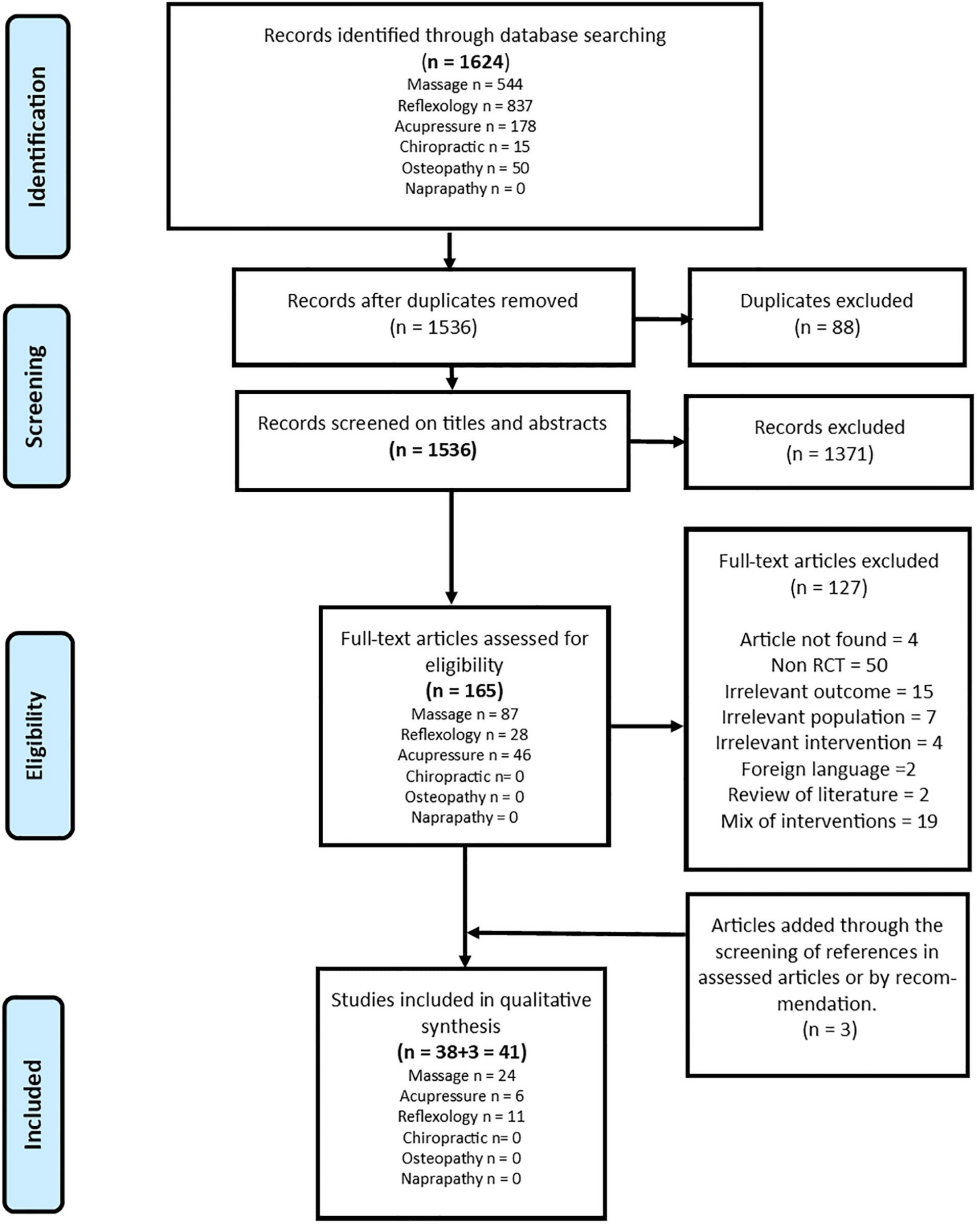
Flow diagram of included studies.

A total of 1,624 citations were found by our search strategy, including 526 through PsycINFO, PsychArticle, Psychology and Behavorial Sciences Collection and Socindex databases, 695 through CENTRAL and 403 through PubMed (Fig 1). After excluding duplicates (n = 88), 1536 records were screened (titles and abstracts), which led to the exclusion of 1371 studies. Thus, 165 full-text articles were assessed, of which 127 were excluded (see Fig 1 for explanations), leaving 38 studies. A further three were added after screening the references of the reviewed articles giving a total of 41 studies for final qualitative synthesis.

### Overall summary

Over half of the studies related to massage (Table 1). Included trials were mainly led in high income countries (European or North American countries, n = 24) and in low income countries (Asia, n = 17). A total of 3044 participants were included across all studies. The median Jadad score was 2 out of 5 (range from 0 to 5), implying inconstant quality of design and high risk of bias in methods across studies. Most studies failed to present double blinding and adequate randomization. As several studies showed both significant and non-significant results, or included several of the selected outcomes (symptoms, mood and QOL), numbers given below are naturally unbalanced. Significant symptom improvement (fatigue, pain, sleep, etc.) after intervention was observed in 25 of the 29 studies measuring symptom outcomes. Balanced results were observed for mood outcomes with 14 of the 24 studies showing significant improvements. Finally, 10 of the 15 studies about quality of life did not find any significant differences in quality of life after interventions (Table 1). It was possible to calculate effect size for 18 studies. Of these papers, 16 studies showed “problematic” effect sizes before the intervention, supporting the reliability of the intervention evaluation by assessing the lack of between-group effects before intervention. After intervention, 15 out of 18 studies showed a significant non-problematic effect. Across all interventions, the largest effect sizes were found on symptoms. Indeed, 12 effect size indicators were available after calculation, and 8 of them were large (≥ 0.8), 3 were moderate (≥0.5) and 1 was small (≥ 0.2) (Tables 2, 3 and 4).

**Table 1:** Reviewed studies in manipulative body-based practices according to cancer, sample, method and outcomes.

	N	Cancer (%)	Sample			Blinding (n/N)	Randomization (n/N)	Jadad Median (Range)	Outcomes*	
			Total	M (SD)	Range				Significant (n/N)	Ns. (n/N)
Overall	41	Breast (34) Mixed (49) Leukemia (7) Digestive (10)	3044	74 (69)	12–348	N/A (21/41) Single (12/41) Double (8/41)	N/A (11/41) Simple (15/41) Block (12/41) Minimized (3/41)	2 (0–5)	Symptom (25/29) QOL (8/15) Mood (14/24)	Symptom (9/29) QOL (10/15) Mood (13/24)
Reflexology	11	Breast (27) Digestive (18) Mixed (55)	998	91 (76)	12–243	N/A (6/11) Single (2/11) Double (3/11)	N/A (4/11) Simple (2/11) Block (3/11) Minimized (2/11)	2 (0–5)	Symptom (7/8) QOL (5/7) Mood (2/5)	Symptoms (2/8) QOL (4/7) Mood (3/5)
Massage Therapy	24	Breast (42) Leukemia (4) Mixed (50) Digestive (4)	1584	66 (74)	21–348	N/A (13/24) Single (9/24) Double (2/24)	N/A (7/24) Simple (11/24) Block (5/24) Minimized (1/24)	2 (1–5)	Symptom (15/16) QOL (3/8) Mood (9/16)	Symptom (4/16) QOL (6/8) Mood (10/16)
Acupressure	6	Breast (17) Leukemia (33) Gastric (17) Mixed (33)	462	77 (23)	43–100	N/A (2/6) Single (1/6) Double (3/6)	N/A (0/6) Simple (2/6) Block (4/6) Minimized (0/6)	3,5 (2–5)	Symptom (3/5) QOL (0) Mood (3/3)	Symptoms (3/5) QOL (0) Mood (0/3)

Note. N/n = number of studies; M = Mean; SD = Standard Deviation; Ns = non-significant; N/A = Not Available.

*Several studies both showed significant and non-significant results, explaining the unbalanced numbers.

**Table 2:** Details of reviewed studies about massage therapy, according to cancer, sample, method design, size effect and outcomes.

Setting			Method		Size effect (Treatment vs Control)				Outcomes	
Ref.	Cancer	N	Jadad	Control strategy	SMD		95% CI		Significant	Ns.
					Before	After	Lower	Upper		
Weinrich, 1990 [37]	Mixed	28	1	Visit by staff	**0.8**	0.3 †	[-0.6	1.3]	Pain (male)	Pain (female)
Wilkie, 2000 [38]	Mixed	29	3	Control	N/A	N/A	N/A	N/A	Pain	QOL
Hernandez-Reif, 2004 [39]	Breast	34	2	Control	0.4 †	**-0.5**‡	[-1.1	-0.1]	Anxiety	Vigor
					**0.9**	**-0.6**‡	[-1.1	-0.1]	Depression	
Soden, 2004 [40]	Mixed	36	3	Massage or Control	N/A	N/A	N/A	N/A	Sleep	Mood, Pain
Wilcock, 2004 [41]	Mixed	29	2	Control	N/A	N/A	N/A	N/A	N/A	Mood, Symptoms, QOL
Billhult, 2007 [42]	Breast	39	1	Visit by staff	N/A	0.7	[0.09	1.4]	Nausea	Anxiety, Depression
Campeau, 2007 [43]	Mixed	100	0	Control	N/A	N/A	N/A	N/A	N/A	Anxiety
Wilkinson, 2007 [44]	Mixed	221	4	Control	N/A	N/A	N/A	N/A	Anxiety, Depression	N/A
Billhult, 2008 [45]	Breast	22	1	Visit by staff	N/A	N/A	N/A	N/A	N/A	Mood, Satisfaction
Kutner, 2008 [46]	Mixed	348	3	Simple touch	N/A	N/A	N/A	N/A	Pain, Mood	Symptoms, QOL
Listing, 2009 [47]	Breast	62	1	Control	-0.2 †	**0.5**‡	[0.2	0.9]	Pain	QOL
					**-0.4**	**-0.3**‡	[-0.7	-0.2]	Symptoms	
					N/A	N/A	N/A	N/A	Mood	
Listing, 2010 [48]	Breast	29	1	Control	0.4 †	-0.4 †‡	[-0.8	0.1]	Anger	Stress
					0.07 †	**-0.8**	[-1.3	-0.3]	Tiredness	
					0.1 †	-0.4 †‡	[0.9	0.02]	Anxious	
Krohn, 2010 [49]	Mixed	29	1	Control	N/A	N/A	N/A	N/A	Depression	Mood, Stress
Fernandez-Lao, 2011 [50]	Breast	20	2	Usual care + attention	0.2 †	-0.3 †	[- 1.2	0.5]	Fatigue	Depression
					- 0.05 †	-0.6 †	[- 1.5	0.3]	Mood	
Jane, 2011 [51]	Mixed	72	5	Visit by staff	0.2 †	**-0.7**‡	[- 1	-0.4]	Pain	N/A
					0.2 †	**-0.4**‡	[-0.6	-0.1]	Mood	
					0.03 †	**0.5**‡	[0.2	0.8]	Relaxation	
					-0.2 †	0.05 †‡	[-0.2	0.3]	Sleep	
Lai, 2011 [52]	Mixed	32	1	Control	N/A	N/A	N/A	N/A	Constipation	QOL
Thot, 2013 [53]	Mixed	39	1	Visit or Control	N/A	N/A	N/A	N/A	QOL	Anxiety, Alertness
Kashani, 2014 [54]	Breast	57	2	Control	0.1 †	**-0.9**	[-1.4	-0.3]	Sleep	N/A
Darabpour, 2016 [55]	Breast	100	1	Control	0.1	**-1.1**‡	[-1.5	-0.7]	Mood	N/A
Donoyama, 2016 [56]	Mixed	40	3	Visit	N/A	N/A	N/A	N/A	Physical complaints	N/A
Miladinia, 2016 [57]	Leukemia.	60	1	Control	N/A	N/A	N/A	N/A	Symptoms	N/A
Kinkead, 2017 [58]	Breast	57	3	Light touch or Control	N/A	N/A	N/A	N/A	Fatigue, QOL	N/A
Ayik, 2018 [59]	Colorectal	80	2	Control	-0.04†	**-1.2**	[-1.7	-0.7]	Sleep	N/A
					-0.31†	**1.3**	[0.8	1.7]	Anxiety	
Massiling, 2018 [60]	Breast	21	2	Relaxation massage	N/A	N/A	N/A	N/A	Pain, Mobility, QOL	N/A

Note. Only the confidence interval of the size effect after intervention is indicated; Size effect in bold are significant; Ref. = Reference; SMD = Standardized Mean Difference; CI = Confidence Interval; N/A = Not available; QOL = Quality of life; Ns. = Non-significant.;

^†^ = “problematic” size effects, as confidence intervals include the value “0” in their range, which means the “statistical” absence of effect;

^‡^ = involved study measured the same variable several times, and a pooled d was obtained by the mean of a meta-analysis with model of random effects via the software ESCI [35].

**Table 3:** Details of reviewed studies about reflexology, according to cancer, sample, method, design, size effect and outcomes.

	Setting		Method		Size effect (Treatment vs Control)				Outcomes	
Ref.	Cancer	N	Jadad	Control strategy	SMD		95% CI		Significant	Ns.
					Before	After	Lower	Upper		
Hogson, 2000 [61]	Mixed	12	2	Placebo Reflexology	N/A	N/A	N/A	N/A	QOL	N/A
Ross, 2002 [62]	Mixed	17	3	Foot massage	N/A	N/A	N/A	N/A	N/A	Symptom, Mood
Stephenson, 2003 [63]	Mixed	36	2	Control	N/A	N/A	N/A	N/A	Pain	N/A
Stephenson, 2007 [64]	Mixed	86	1	Control	N/A	N/A	N/A	N/A	Pain, Anxiety	N/A
Tsay, 2008 [65]	Digestive	61	5	Control	-0.1 †	**- 1**‡	[-1.2	-0.7]	Pain	N/A
					-0.3 †	**- 1.2**	[-1.7	-0.6]	Anxiety	
Sharp, 2010 [66]	Breast	183	3	Scalp massage or Control	N/A	N/A	N/A	N/A	Relaxation	Mood, QOL
Wyatt, 2012 [67]	Breast	243	4	Lay foot manipulation or Control	0.01 †	0.2 †‡	[0.02	0.4]	Physical	QOL, Mood, Pain, Nausea
					N/A	N/A	N/A	N/A	Fatigue	
Ozdelikara, 2017 [68]	Mixed	60	1	Control	0.9 †	**-2.5**	[-3.2	-1.9]	Symptom, QOL	N/A
					-0.6 †	**2**	[1.3	2.6]		
					0.1 †	**2.9**	[2.2	3.6]		
Uysal, 2017 [69]	Colorectal	60	2	Classical massage or Control	N/A	N/A	N/A	N/A	Pain	N/A
					N/A	N/A	N/A	N/A	Fatigue	
					0.3 †	0.05 †‡	[-0.3	0.4]	Symptoms	
					**0.7**	**1.1**‡	[0.7	1.6]	Physical	
Wyatt, 2017 [70]	Breast	180	4	Control	N/A	N/A	N/A	N/A	Symptom	Function, Satisfaction
Kurt, 2018 [71]	Mixed	60	1	Control	-0.1 †	**-0.6**‡	[-1	-0.3]	Function	Neuropathy

Note. Only the confidence interval of the size effect after intervention is indicated; Size effect in bold are significant; Ref. = Reference; SMD = Standardized Mean Difference; CI = Confidence Interval; N/A = Not available; QOL = Quality of life; Ns. = Non-significant.;

^†^ = “problematic” size effects, as confidence intervals include the value “0” in their range, which means the “statistical” absence of effect;

^‡^ = involved study measured the same variable several times, and a pooled d was obtained by the mean of a meta-analysis with model of random effects via the software ESCI [35].

**Table 4:** Details of reviewed studies about acupressure, according to cancer, sample, method design, size effect and outcomes.

Setting			Method		Size effect (Treatment vs Control)				Outcomes	
Ref.	Cancer	Sample	Jadad	Control strategy	SMD		95% CI		Significant	Ns.
					Before	After	Lower	Upper		
Beikmoradi, 2015 [72]	Mixed	85	5	Sham or Control	-0.09 ††	**0.9**‡	[-1.3	-0.4]	Anxiety St	N/A
					0.1	0.1 †‡	[- 0.5	0.2]	Anxiety Tr	
Hsiung, 2015 [73]	Gastric	54	2	Control	N/A	N/A	N/A	N/A	Pain	Nausea
Avci, 2016 [74]	Leukemia	90	2	Band wrist	N/A	N/A	N/A	N/A	N/A	Nausea
Nia, 2017 [75]	Leukemia	100	3	Control	N/A	N/A	N/A	N/A	N/A	Pain
Rizi, 2017 [76]	Mixed	90	5	Sham or Control	0.3 †	**- 1.6**	[-2.2	-1]	Pain	N/A
					0.2 †	**- 0.5**	[- 1.1	-0.04]	Anxiety	
Zhang, 2017 [77]	Breast	43	4	Sham	N/A	N/A	N/A	N/A	Fatigue, Mood, Sleep	N/A

Note. Only the confidence interval of the size effect after intervention is indicated; Size effect in bold are significant; Ref. = Reference; SMD = Standardized Mean Difference; CI = Confidence Interval; N/A = Not available; QOL = Quality of life; Ns. = Non-significant.;

^†^ = “problematic” size effects, as confidence intervals include the value “0” in their range, which means the “statistical” absence of effect;

^‡^ = involved study measured the same variable several times, and a pooled d was obtained by the mean of a meta-analysis with model of random effects via the software ESCI [35].

### Massage therapy results (n = 24)

Massage studies have been mainly led in high-income countries (United States n = 8; Germany n = 3; United Kingdom n = 2; Sweden n = 2; Quebec n = 1; Spain n = 1), and fewer in Asian countries (Iran n = 3; Turkey, Japan, China and Taiwan n = 1 each one). Mean age of participants varied from 34.5 to 71.5 (M = 55.9, SD = 7.9). 70% of the studies included mostly female participants (50% female-only participants, 20% more than half participants). In these studies, only six used a control strategy which featured a “placebo” intervention (a simple massage or touch) or used more than two groups. Thirteen studies featured mainly a simple control group/ usual care (54%), and five an attention group “visit by staff” (23%). Interventions were delivered by a professional therapist in 58% of the studies, while 30% of the interventions were delivered by trained nurses or authors. Number of interventions ranged from 1 to 15 sessions (M = 7.1, SD = 4.5), and length varied from 10 to 60 minutes each session (M = 29.8, SD = 12.9). Details of these studies can be found in Table 2.

Fifteen of the 24 studies showed an improvement in symptoms in the intervention group. Nevertheless, most of these studies had small samples and a Jadad score of 0 to 3. The most trustworthy trial, with a Jadad score of 5, showed improvements across time in pain levels, both clinically and statistically (F = 61.17, p = 0.000) [51].

Concerning mood outcomes, four studies showed a significant decrease in anger, anxiety, depression, stress and mood disturbance (anger, anxious depression) in people receiving massage compared to the control group [39; 48, 50, 55]. Nevertheless, these previous studies feature a high risk of bias. A robust study on patients with breast cancer showed that patients receiving massage therapy had a significant improvement in clinical anxiety and depression at six weeks post randomization [44].

### Reflexology results (n = 11)

In reflexology studies (n = 11), the mean age of the participants ranged from 51 to 74 (M = 59.2, SD = 6.1). As for massage studies, they were mainly led in high-income countries (United Sates n = 4, United Kingdom n = 3 vs. Turkey n = 3 and Taiwan n = 1). 81% of the studies included mostly female participants (36% female-only participants, 45% more than half participants). Five studies featured at least a placebo intervention like sham reflexology or more than two groups (one “placebo” intervention and one control group/usual care). Six studies featured control group only. Interventions were delivered by reflexologists in 7 on11 studies, by trained authors in 2. Number of sessions ranged from 2 to 84 (M = 12.4, SD = 23.9) and length varied from 20–60 minutes (M = 32.5, SD = 11.4).

Seven studies showed improvement on symptoms, although most had a high risk of bias (Jadad score ranging from 1 to 2). Both studies of Wyatt et al. carried strong randomized controlled trials evaluating reflexology for breast cancer patients (large sample size, Jadad score of 4). The first one [66] focused on breast cancer (N = 243) comparing reflexology to lay foot manipulation and a control group with three waves of data collection (prior to intervention, 1 week after intervention and six weeks after the intervention). No differences were found on pain and nausea, but a significant reduction in dyspnea severity was found compared to lay foot manipulation group (p = .02) and control group (p < .01). The second one found a clearer and stronger significant decrease of symptom severity (p = .02) and symptom interference (p < .01) over the 11 weeks of the trials in the reflexology group compared to the control group (N = 180). These symptoms include pain, fatigue, nausea, disturbed sleep, distress, shortness of breath, difficulty remembering, decreased appetite, drowsiness, dry mouth, sadness, vomiting and numbness/tingling. A final study [65] with a Jadad score of 5 (N = 61) demonstrated that digestive cancer patients in the intervention group reported less pain over time and that their consumption of opioid analgesics decreased compared to the control group. The same authors also demonstrated a higher decrease in anxiety in the intervention group than in the control group (p < .05). This is one of the only studies that reported a benefit of reflexology on anxiety, with a previously cited study [64]. For more details, see Table 3.

### Acupressure results (n = 6)

Acupressure studies were exclusively carried out in countries in Asia (Iran n = 3; Turkey n = 1; China n = 1; Taiwan n = 1). The mean age of participants varied from 34.5 to 71.5 (M = 51.7, SD = 6.5). Around 50% of the studies featured female participants mainly (1 study on 6 female only-participants, 2 studies on 6 more than half participants. 4 of 6 studies used a control strategy which featured a “sham acupressure” intervention. Interventions were delivered by a professional therapist in only two studies, while the rest of the interventions were led by researchers. Dose of the interventions ranged from 3 to 36 sessions (M = 13, SD = 13.4), and length varied from 8 to 30 minutes each session (M = 17, SD = 9.8).

Three studies argued in favor of a positive effect of acupressure on symptoms, of which one trial with a perfect Jadad score and high number of participants shows a strong effect on pain [76]. All three studies evaluating anxiety highlighted a beneficial effect of acupressure (Table 4).

## Discussion

The aim of this study was to achieve a global understanding of the effects of manipulative and body-based therapies as alternative and integrative therapies in oncology. First of all, although chiropractic and osteopathy are widely used by cancer patients and their use is often recommended on cancer websites [13, 78], our systematic review did not find any trials on chiropractic and osteopathy. This result is consistent with the findings of Alcantar et al. [78] who showed in their systematic review of chiropractics in oncology in 2012 that most papers were based on survey and case discussions only. Further studies are needed to evaluate these practices by randomized trials with cancer patients. Most of the participants in the 41 studies were women. This result is explained by the majority of studies focusing on breast cancer. A systematic review of CAM use in oncology highlights the fact that gender is one of the several dominant characteristics associated with CAM use, with women resorting more to CAM than men [79]. It has also been suggested that women suffer more from chronic illnesses and use healthcare more extensively than men [80]. In that respect, it would be advisable to promote manipulative and body-based practice studies alongside males to gain a more extensive knowledge.

Our review showed that manipulative and body-based practices tended to offer positive effects on cancer symptoms. All four studies with the highest quality methodology (Jadad score = 5) showed large effects on pain and/or anxiety after interventions in reflexology, massage or acupressure [51, 65, 72, 76] but most of the calculated effect sizes above were pooled from different measures of time, denying arbitrarily the possibility of a delayed or cumulated effect over time. Pain and fatigue seemed to be the main symptoms positively affected by the interventions. Assumptions about the mechanisms underlying the efficacy of massage or reflexology range from biological explanation (increase of blood flow and lymph, influence of the autonomic nervous system, stimulation of release of endogenous opiates) to energy-based explanations (removing energy blockages, ensuring recirculation in the blocked areas, increase in the energy level) [31, 39, 65, 69]. Nevertheless, as these practices are based on touch, it may be hard to determine whether the therapeutic effect is linked to the specificities of the practice or to the attention through human touch and massage-like manipulation [23].

Unfortunately, less than half of the studies used a placebo group to assess the interventions. This is most certainly due to the cost of implementing placebo versus intervention designs, as well as the difficulties in designing adequate and convincing placebo interventions of manual therapies. In addition, our findings show the lack of convincing method, as most of the studies fail to report adequate blinding and randomization. This is consistent with previous reviews of literature evaluating body-based practices [15,18–22, 81–83], which suggested that even though most of the studies tend to find positive effects of massage or reflexology on symptoms in cancer, the heterogeneity of methods, the small sample sizes and the high risk of bias confound the reading of the results and prevent us from drawing a clear interpretation. As such, these previous reviews called for supplementary studies with stronger methods to reduce bias. Recent studies have flourished [55–60, 68–71, 74–77], and yet their methodologies are still affected by a high risk of bias, showing the difficulties involved in leading strong RCTs with non-pharmaceutical interventions. Further randomized controlled trials are still needed to clear remaining doubts, and these trials should be supplied with large samples, adequate randomization and appropriate blinding. Ideally, those trials should be multi-centric, and tested interventions should be administered by several different practitioners, which would be a more truthful representation of CAM.

On another hand, even though RCT is arguably the most powerful study design to assess the efficacy of an intervention, there is still some limits to their uses. Research on chronic diseases is already complex as participants are weakened, difficult to access, and have a high risk of attrition. The high cost of RCTs is well known too: a strict approach is necessary to respect the reproducibility of the results. The price is a high cost of time, funds, and severe criteria of inclusion/exclusion that sacrifice external validity for internal validity [84]. Thus, different methods of appraisal may be beneficial and complementary to RCTs. Price et al. suggest resorting to “real life” studies when RCTs show their limits [84]. Thus, observational studies (cross-sectional, cohort,) and pragmatic trials (real-life context of clinic) could prove complementary when assessing questions that are “unanswered or unanswerable” by RCT, especially in CAM [85]. Another interesting experimental design is the single-case research design. According to Smith’s literature review of the single-case design, this is one of the most cost-effective and efficient procedures to experimentally test the effect of an intervention [86]. Such design, with adequate replication across different participants and different locations, could prove to be a valuable asset in evaluating an intervention.

Finally, the result of this review must be discussed in the context of a recent observational cohort study that found that cancer patients who received CAM were more likely to refuse or delay conventional treatment and to have a two-fold higher risk of death, making use of CAM a mortality risk [87]. Although the authors mention several limitations (survival difference could be mediated by adherence to conventional therapies, inclusion of many different kind of CAM prevents the possibility to discuss of any specific type of CAM), their study highlights the importance of caution and robust methodology when researching or recommending CAM.

### Clinical implications

We believe that this review provides a starting point for a specific and sound discussion about manipulative CAM on cancer patients, as it seems that they could be beneficial for symptoms management in cancer. This study could help physicians, oncologists and their patients make informed choices about their care. Nevertheless, no study evaluated differences in survival rates with patients using this specific kind of CAM and doubts persist. In that respect, we advise that patients with cancer who are interested in receiving this type of CAM should use them in a complementary way, discuss about it with their physicians and be warned of the lethal dangers of neglecting conventional treatments.

### Study limitations

Our findings should be considered keeping in mind several limitations. Firstly, although we investigated the use of CAM in all cancers, most of the studies only included breast cancer. Secondly, we tried to discriminate results by practice. However, the subgroup of massage therapy studies had high heterogeneity, as it included different methods of massage therapy. Sagar, Dryden and Wong [31] discriminate massage technique by two main branches, including many kinds of massage (Swedish, Trigger point, Neuromuscular, Craniosacral therapy). In our work, we did not go to such lengths and included all massages in the same group. Another limitation is that we did not register our protocol on the database PROSPERO. Unfortunately, our data extraction process was already finished by the time we learnt about PROSPERO. Another limit lies in the tool we used to assess the quality of trials. Although the Jadad scale is one of the most used quality assessment scale, it is also a scale that is considered lacking (over-simplistic, too much emphasize on blinding, low agreement between raters, no assessment of allocation concealment) [88, 89]. As such, future researches evaluating RCTs should rely on more solid, complete and recent assessment tool, as the Cochrane risk-of-bias tool for randomized trials (RoB 2) [90, 91]. Moreover, we did not carry out meta-analysis because of the high heterogeneity of included studies, and thus crucial information are missing, such as forest plot, publication bias, aggregated effect size, and more importantly subgroup analysis that could have given precious details on which intervention would work better on which outcome. Although no formal meta-analysis was possible, additional tools could have been used to strengthen our study, such as the albatross plot, which is designed for the report of diversely reported studies [92]. Unfortunately, this tool is relatively new and only accessible yet on the software STATA. No studies about osteopathy, chiropractic or naprapathy could be found, preventing us to draw an exhaustive conclusion on Manipulative and Body-Based Practice. On another note, the process of selection, inclusion/exclusion of papers, and data extraction/comparison was only semi-dual and independent, as it was performed on the first 20 studies of each databases. Even if consensus was easily reached between the two independent collectors, there are still risks that the selection of some studies and data extraction may have been biased, as the process was not fully independently verified by both collectors. Finally, this systematic review is incomplete, in that we could not access to the full text of 4 identified articles.

## Conclusion

Of 41 studies, including 29 of which assess symptoms, a significant improvement of symptoms was found in 25 studies. The evidence for improvements in cancer symptom management was stronger than for improvements in mood or quality of life. Nevertheless, heterogeneity and weaknesses in the methods used prevents any firm conclusions and cancer patients interested in CAM should be advised to use them responsibly and avoid stopping conventional treatment for their own safety. Further trials are needed to gain a comprehensive knowledge of their effects. These studies will require robust methodology that is onerous and difficult to attain, requiring substantial financial support over time. More rigorous RCTs are needed, and other experimental designs that could prove to be complementary are available.

## Supporting information

S1 File Data file of the study. This table contains all the data used for the narrative review.
(XLSX)

S2 File PRISMA document. This file contains the references of the pages according to PRISMA Statement.
(DOC)
